# Social Isolation and its Brain Correlations: From Symptomatology to Neuroimaging Findings

**DOI:** 10.1192/j.eurpsy.2022.103

**Published:** 2022-09-01

**Authors:** M. Bellani

**Affiliations:** University of Verona, Department Of Neurosciences Biomedicine And Movement Sciences, Verona, Italy

**Keywords:** social isolation, brain networks, Brain, Neuroimaging

## Abstract

According to the social brain hypothesis, the human brain includes a network designed for the processing of social information. This network includes several brain regions that elaborate social cues, interactions and contexts, i.e. prefrontal paracingulate and parietal cortices, amygdala, temporal lobes and the posterior superior temporal sulcus. We will explore neuroimaging studies that investigated social isolation in healthy subjects.

**Disclosure:**

No significant relationships.

